# Serum potassium as a predictor of adverse clinical outcomes in patients with chronic kidney disease: new risk equations using the UK clinical practice research datalink

**DOI:** 10.1186/s12882-018-1007-1

**Published:** 2018-08-22

**Authors:** Hans Furuland, Phil McEwan, Marc Evans, Cecilia Linde, Daniel Ayoubkhani, Ameet Bakhai, Eirini Palaka, Hayley Bennett, Lei Qin

**Affiliations:** 1 Department of Nephrology, Uppsala University Hospital, Uppsala, Sweden; 2 Health Economics and Outcomes Research Ltd, Cardiff, UK; 3 School of Human and Health Sciences, Swansea University, Swansea, UK; 4 Diabetes Resource Centre, University Hospital Llandough, Cardiff, UK; 5 Heart and Vascular Theme, Karolinska University Hospital and Karolinska Institutet, Stockholm, Sweden; 6 Department of Cardiology, Royal Free Hospital, London, UK; 7 Global Health Economics, AstraZeneca, Cambridge, UK; 8 Global Health Economics, AstraZeneca, 101 Orchard Ridge Drive, Gaithersburg, MD 20878 USA

**Keywords:** Hyperkalaemia, Serum potassium; chronic kidney disease, Mortality, Major adverse cardiac event, RAASi discontinuation

## Abstract

**Background:**

To address a current paucity of European data, this study developed equations to predict risks of mortality, major adverse cardiac events (MACE) and renin angiotensin-aldosterone system inhibitor (RAASi) discontinuation using time-varying serum potassium and other covariates, in a UK cohort of chronic kidney disease (CKD) patients.

**Methods:**

This was a retrospective observational study of adult CKD patients listed on the Clinical Practice Research Datalink, with a first record of CKD (stage 3a–5, pre-dialysis) between 2006 and 2015. Patients with heart failure at index were excluded. Risk equations developed using Poisson Generalized Estimating Equations were utilised to estimate adjusted incident rate ratios (IRRs) between serum potassium and adverse outcomes, and identify other predictive clinical factors.

**Results:**

Among 191,964 eligible CKD patients, 86,691 (45.16%), 30,629 (15.96%) and 9440 (4.92%) experienced at least one hyperkalaemia episode, when defined using serum potassium concentrations 5.0–< 5.5 mmol/L, 5.5–< 6.0 mmol/L and ≥ 6.0 mmol/L, respectively. Relative to the reference category (4.5 to < 5.0 mmol/L), adjusted IRRs for mortality and MACE exhibited U-shaped associations with serum potassium, with age being the most important predictor of both outcomes (*P* < 0.0001). A J-shaped association between serum potassium and RAASi discontinuation was observed; estimated glomerular filtration rate was most predictive of RAASi discontinuation (*P* < 0.0001).

**Conclusions:**

Hyperkalaemia was associated with increased mortality and RAASi discontinuation risk. These risk equations represent a valuable tool to predict clinical outcomes among CKD patients; and identify those likely to benefit from strategies that treat hyperkalaemia, prevent RAASi discontinuation, and effectively manage serum potassium levels.

**Electronic supplementary material:**

The online version of this article (10.1186/s12882-018-1007-1) contains supplementary material, which is available to authorized users.

## Background

Hyperkalaemia, typically defined as serum potassium concentration exceeding 5.0 mmol/L, is a potentially life-threatening electrolyte imbalance [1]. Recent epidemiological studies have consistently demonstrated associations between hyperkalaemia and adverse clinical outcomes; notably hospitalisation, cardiovascular morbidity and mortality [2–6].

As a consequence of impaired renal function, patients with chronic kidney disease (CKD) are at increased risk of hyperkalaemia; and renin-angiotensin-aldosterone system inhibitor (RAASi) agents routinely indicated for CKD management [1, 7] are known to exacerbate this risk [8]. Despite this, the current practice of down-titrating or discontinuing guideline-recommended RAASi therapy in response to acute hyperkalaemia [9, 10] has recently been associated with worsening clinical outcomes and greater total costs in patients with CKD or other comorbidities [11–14].

Real-world studies associating serum potassium and adverse clinical outcomes in CKD patients, including death, major adverse cardiac events (MACE), hospitalisation and RAASi discontinuation, have previously been conducted in the US [2–6]. In contrast to other industrialised countries, healthcare in the US is largely privatised, and the availability of clinical data may be limited by the size, length and claims-based nature of US healthcare records. Thus, it is unclear whether existing data describing the epidemiology and burden of hyperkalaemia may be generalisable to a European CKD population. Using primary care data obtained from the Clinical Practice Research Datalink (CPRD) [15], this study sought to develop risk equations describing the relationship between serum potassium concentration and incidence of death, MACE and RAASi discontinuation, in a contemporary UK cohort of CKD patients.

## Methods

### Study data and patient population

This retrospective analysis utilised data from the CPRD and linked Hospital Episode Statistics (HES). The CPRD is an observational research database comprised of anonymised, longitudinal primary care records for approximately 7% of the UK population. It is broadly representative of the general population, and is the world’s largest computerised database of medical records suitable for public health research [16]. Linked HES data described admissions, outpatient appointments, and emergency episodes in National Health Service hospitals in England [17]. The study was approved by the Independent Scientific Advisory Committee for Medicines and Healthcare Products Regulatory Agency database research on 15 December 2016 (study protocol 16_223R).

Inclusion criteria for the study were adult CKD patients (aged ≥18 years) in the CPRD between 1 January 2006 and 31 December 2015, who experienced a first record of CKD (stage 3a to 5, pre-dialysis; herein referred to as the index date) after the study start date. Patients were selected on the basis of estimated glomerular filtration rate (eGFR; defined as < 60 mL/min/1.73m^2^ [7]), Read codes (CPRD), and ICD-10 codes (HES). Exclusion criteria comprised patients on dialysis at index date or with a CKD event prior to 1 January 2006, and patients with a history of heart failure (HF; identified by Read and ICD-10 codes) at index date, due to the increased risk of hyperkalaemia associated with RAASi usage [9]. Read and ICD-10 codes were further used to define clinical outcomes and covariates (Additional file 1: Table S1).

### Study design and data structuring

The clinical outcomes studied in this analysis included all-cause mortality, incidence of MACE (myocardial infarction, arrhythmia, HF, and stroke), and discontinuation of RAASi medications. Using the medicines possession ratio, RAASi discontinuation was defined as the first 90-day gap after the estimated end-date of a RAASi prescription. Agents comprising RAASi therapy included angiotensin-converting enzyme (ACE) inhibitors, angiotensin II receptor blockers (ARBs), mineralocorticoid receptor antagonists (MRAs), and renin inhibitors. Hyperkalaemia was defined according to serum potassium intervals of ≥5.0 mmol/L to < 5.5 mmol/L, ≥5.5 mmol/L to < 6.0 mmol/L, and ≥ 6.0 mmol/L. Serum potassium < 3.5 mmol/L was classified as hypokalaemia.

Serum potassium was time-updated during the follow-up period; an illustrative example of this methodology is provided in Additional file 1 and Additional file 2: Figure S1. In brief, each patient’s most recent serum potassium measurement was applied until a new measurement became available, using a last-observation-carried-forward (LOCF) approach [4]. Further, time-updated eGFR readings were the most recently available at the time of each serum potassium measurement. The period between serum potassium measurements defined patient-intervals, and clinical events of interest were assigned to these based on the date on which they occurred.

Time-updated serum potassium was evaluated categorically (< 3.5, 3.5 to < 4.0, 4.0 to < 4.5, 4.5 to < 5.0, 5.0 to < 5.5, 5.5 to < 6.0, and ≥ 6.0 mmol/L). Exposure time in each serum potassium category was quantified in patient-years. Patients were followed up to and including the first occurrence of loss to follow-up, end of study period (31 December 2015), or death.

### Statistical analyses

The data were analysed using R version 3.3.2 [18]. Means, medians, counts and/or proportions, and associated measures of variability were used to describe patient demographics. Patient characteristics at index date were used to calculate summary statistics; baseline clinical measurements were defined as the first measurement taken in the 3 months following the index date. Disease history was analysed over the 5 years prior to the index date and was expressed using counts and proportions. Medication usage at baseline was defined as having at least one prescription during a six-month period centred around the index date and expressed as counts and proportions. Missing values were not included in calculations relating to baseline patient characteristics, disease history and medication usage.

Hyperkalaemia episodes of increasing severity were defined as serum potassium measurements within each interval (≥5.0 mmol/L to < 5.5 mmol/L, ≥5.5 mmol/L to < 6.0 mmol/L, and ≥ 6.0 mmol/L), without a hyperkalaemic measurement of at least that severity (serum potassium ≥5.0 mmol/L, ≥5.5 mmol/L, and ≥ 6.0 mmol/L, respectively) in the preceding 7 days. Subsequently, hyperkalaemia episodes were assumed to persist for a maximum duration of 1 week. The median time between successive episodes was calculated among only those patients who experienced the given number of episodes; censoring due to death, loss to follow-up or end of study period was not taken into account.

The incidence of each outcome (death, MACE and RAASi discontinuation) was predicted using a risk equation obtained by fitting a statistical model to the event count in each patient-interval. Risk equations were estimated using Generalized Estimating Equations with an exchangeable working correlation structure to account for intra-patient correlation. Events were assumed to be Poisson distributed. Risk equations included a natural logarithm link function and an offset equal to the natural logarithm of patient-years (defined as the exposure time in each patient-interval). Patients with a serum potassium of 4.5 to < 5.0 mmol/L were used as the reference group for comparison, from which incident rate ratios (IRRs) were estimated.

To address the possibility of long durations between measurements of serum potassium and the incidence of death, MACE and RAASi discontinuation, adjusted IRRs were re-estimated after restricting patient-intervals to a maximum of 30 days post-potassium measurement. Additional sensitivity analysis explored the relationship between CKD stage and observed associations between serum potassium and clinical outcomes, by estimating adjusted IRRs in CKD patients stratified according to time-updated eGFR: 45 to < 60 mL/min/1.73m^2^ (CKD 3a); 30 to < 45 mL/min/1.73m^2^ (CKD 3b); 15 to < 30 mL/min/1.73m^2^ (CKD 4); < 15 mL/min/1.73m^2^ (CKD 5).

Multiple imputation was used to inform missing baseline measurements when estimating the risk equations, which were carried forward using LOCF. Five multiply imputed datasets were produced; model coefficients and their standard errors were pooled across datasets using Rubin’s Rules [19], in order to capture the variance of the coefficients both within and between the imputed datasets. Using the method of Chained Equations, as implemented in the R package ‘mice’ [18], multiple imputation was performed on all clinical variables with all candidate covariates and outcome variables from the analysis models included in the imputation models.

Model assessment and final selection was informed by the Quasi-Likelihood Information Criterion on a randomly selected training sub-sample, and the prediction mean squared error on a randomly-selected validation sub-sample [20]. Predicted incidence rates and IRRs were adjusted for covariates, included as explanatory variables in the risk equations. Adjusted IRRs relating serum potassium to the incidence of mortality and MACE estimated by the fitted risk equations were validated against those published by Luo et al. [4]: a real-world study of CKD stage 3+ patients of similar design to the present study, but using data collected in the US.

## Results

### Patient demographics and characteristics at baseline

**Fig. 1:**
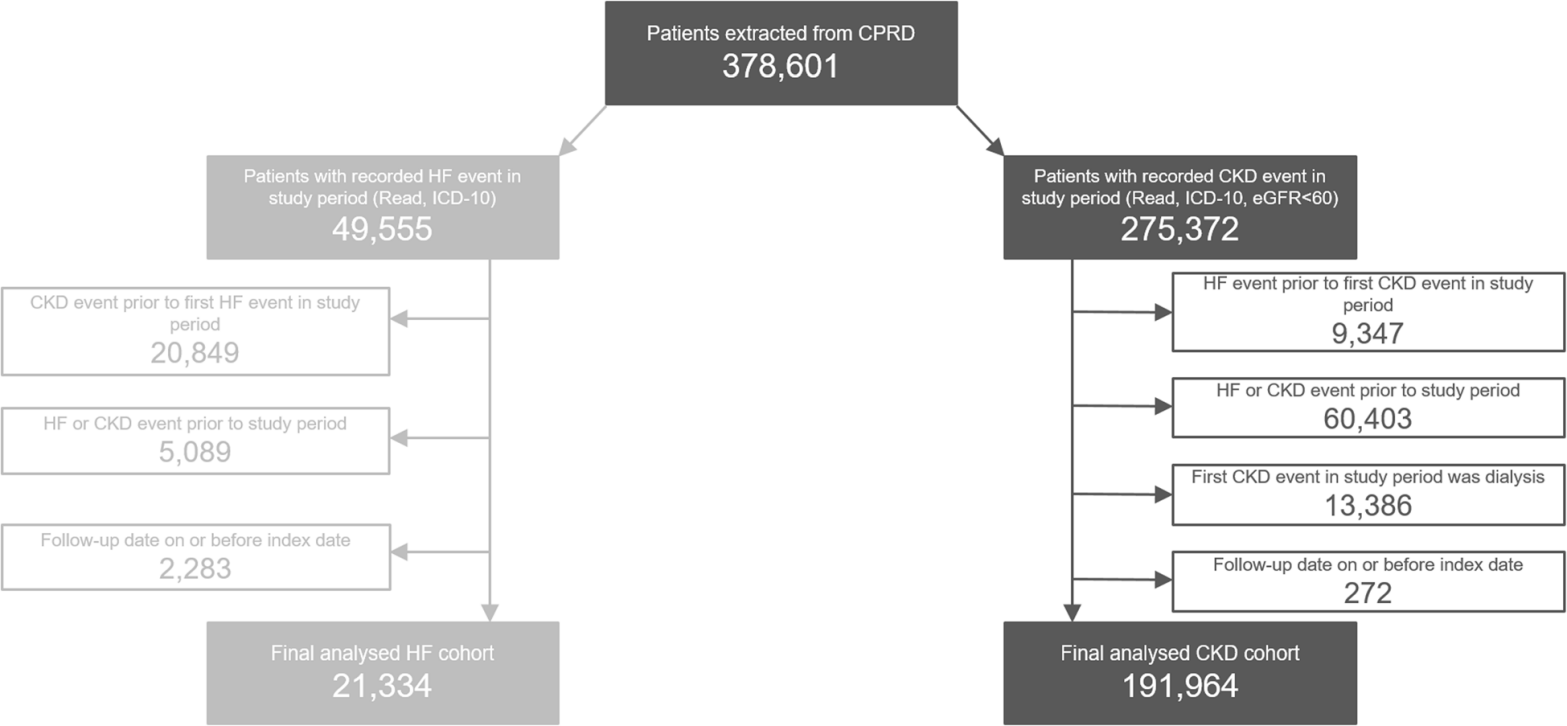
Study participation flow diagram**.** *CKD: chronic kidney disease; CPRD: Clinical Practice Research Datalink; eGFR: estimated glomerular filtration rate; HF: heart failure; ICD: International Classification of Diseases*

The analysed cohort consisted of 191,964 eligible CKD patients, with a mean follow-up time of 4.96 years (Fig. 1; Table 1). At baseline, the mean age was 72.48 years, the majority of the cohort was female (60.61%), and diabetes was the most prevalent comorbidity (14.63%). Based on the earliest measurement recorded in the first 3 months post-index, mean eGFR was 50.96 mL/min/1.73m^2^ and mean serum potassium was 4.47 mmol/L. When patients were stratified by serum potassium category at baseline, the proportion of female patients in each subgroup decreased from 68.50% (< 3.5 mmol/L category) to 53.22% (≥6.0 mmol/L category).

**Table 1:** Baseline patient demographics and clinical histories of chronic kidney disease patients, stratified by serum potassium category

Variable	All	Serum potassium at baseline (mmol/L)^a^						
		< 3.5	3.5 to < 4.0	4.0 to < 4.5	4.5 to < 5.0	5.0 to < 5.5	5.5 to < 6.0	≥6.0
Number of patients	191,964	3635	17,662	50,065	48,543	18,454	4250	1026
Follow-up time (years), mean (SD)	4.96 (2.76)	4.65 (3.06)	5.17 (2.86)	5.14 (2.80)	5.02 (2.79)	4.83 (2.81)	4.62 (2.85)	4.25 (2.94)
Patient demographics, mean (SD)^b^								
Age (years)	72.48 (12.26)	73.46 (13.08)	72.45 (12.59)	71.63 (12.39)	72.03 (12.12)	72.53 (11.80)	72.67 (11.87)	71.97 (13.48)
Male, n (%)	75,611 (39.39%)	1145 (31.50%)	6365 (36.04%)	19,000 (37.95%)	19,958 (41.11%)	8118 (43.99%)	1987 (46.75%)	480 (46.78%)
Current smoker, n (%)	27,078 (16.1%)	530 (16.20%)	2511 (15.50%)	7047 (15.31%)	7577 (16.87%)	2987 (17.48%)	762 (19.35%)	192 (20.3%)
Body mass index (kg/m^2^)	28.53 (5.89)	27.80 (6.50)	28.32 (5.92)	28.44 (5.75)	28.69 (5.90)	28.71 (6.05)	28.38 (6.14)	28.41 (7.13)
Systolic blood pressure (mmHg)	139.77 (19.96)	138.87 (21.95)	140.33 (20.24)	140.07 (20.05)	139.82 (19.92)	139.61 (20.09)	139.86 (21.05)	138.42 (23.69)
eGFR (mL/min/1.73m^2^)	50.96 (8.43)	49.93 (8.84)	51.59 (7.63)	51.88 (7.54)	51.14 (8.15)	49.63 (9.45)	47.00 (11.47)	42.17 (14.48)
Serum potassium (mmol/L)	4.47 (0.53)	3.20 (0.24)	3.76 (0.13)	4.22 (0.14)	4.68 (0.14)	5.15 (0.14)	5.64 (0.13)	6.36 (0.51)
Serum phosphorus (mmol/L)	1.14 (0.94)	1.06 (0.24)	1.08 (0.21)	1.13 (1.32)	1.14 (0.21)	1.17 (0.24)	1.23 (0.30)	1.32 (0.39)
Clinical history at baseline, n (%)^c^								
History of diabetes	28,089 (14.63%)	330 (9.08%)	1778 (10.07%)	5658 (11.30%)	7437 (15.32%)	3719 (20.15%)	1025 (24.12%)	284 (27.68%)
History of myocardial infarction	6277 (3.27%)	74 (2.04%)	419 (2.37%)	1433 (2.86%)	1661 (3.42%)	802 (4.35%)	192 (4.52%)	46 (4.48%)
History of stroke	12,087 (6.30%)	251 (6.91%)	1114 (6.31%)	2969 (5.93%)	2875 (5.92%)	1198 (6.49%)	272 (6.40%)	73 (7.12%)
History of arrhythmia	14,835 (7.73%)	313 (8.61%)	1416 (8.02%)	3812 (7.61%)	3767 (7.76%)	1482 (8.03%)	327 (7.69%)	83 (8.09%)
History of peripheral vascular disease	4620 (2.41%)	76 (2.09%)	374 (2.12%)	1057 (2.11%)	1174 (2.42%)	507 (2.75%)	148 (3.48%)	40 (3.90%)
History of chronic pulmonary disease	18,401 (9.59%)	381 (10.48%)	1727 (9.78%)	5003 (9.99%)	4805 (9.90%)	1911 (10.36%)	414 (9.74%)	95 (9.26%)
History of malignancy	17,658 (9.20%)	443 (12.19%)	1700 (9.63%)	4658 (9.3%)	4497 (9.26%)	1814 (9.83%)	404 (9.51%)	122 (11.89%)
History of metastatic tumour	4296 (2.24%)	121 (3.33%)	466 (2.64%)	1243 (2.48%)	1082 (2.23%)	421 (2.28%)	108 (2.54%)	31 (3.02%)
History of rheumatic disease	6377 (3.32%)	155 (4.26%)	784 (4.44%)	1884 (3.76%)	1538 (3.17%)	538 (2.92%)	119 (2.80%)	30 (2.92%)
History of peptic ulcer	1649 (0.86%)	30 (0.83%)	126 (0.71%)	410 (0.82%)	405 (0.83%)	178 (0.96%)	34 (0.80%)	7 (0.68%)
History of dementia	4749 (2.47%)	179 (4.92%)	561 (3.18%)	1235 (2.47%)	1009 (2.08%)	360 (1.95%)	80 (1.88%)	35 (3.41%)
Medication use at baseline, n (%)^d^								
ACE inhibitors	67,886 (35.36%)	1116 (30.70%)	5912 (33.47%)	17,604 (35.16%)	18,859 (38.85%)	7970 (43.19%)	1926 (45.32%)	467 (45.52%)
ARBs	26,517 (13.81%)	482 (13.26%)	2465 (13.96%)	6952 (13.89%)	6850 (14.11%)	2926 (15.86%)	682 (16.05%)	151 (14.72%)
Renin inhibitors	68 (0.04%)	0 (0.00%)	10 (0.06%)	15 (0.03%)	17 (0.04%)	9 (0.05%)	0 (0.00%)	0 (0.00%)
MRAs	4911 (2.56%)	200 (5.50%)	445 (2.52%)	1106 (2.21%)	1330 (2.74%)	744 (4.03%)	281 (6.61%)	103 (10.04%)
Any RAASi therapy^e^	92,267 (48.06%)	1630 (44.84%)	8235 (46.63%)	23,931 (47.80%)	25,102 (51.71%)	10,567 (57.26%)	2566 (60.38%)	613 (59.75%)
CCBs (DHP)	45,640 (23.78%)	1227 (33.76%)	5106 (28.91%)	12,191 (24.35%)	11,294 (23.27%)	4362 (23.64%)	1117 (26.28%)	290 (28.27%)
CCBs (non-DHP)	7568 (3.94%)	148 (4.07%)	798 (4.52%)	2093 (4.18%)	1885 (3.88%)	745 (4.04%)	184 (4.33%)	31 (3.02%)
NSAIDs	26,739 (13.93%)	466 (12.82%)	2453 (13.89%)	7277 (14.54%)	7424 (15.29%)	2993 (16.22%)	695 (16.35%)	159 (15.50%)
Diuretics	77,143 (40.19%)	2842 (78.18%)	10,850 (61.43%)	21,548 (43.04%)	16,819 (34.65%)	6182 (33.5%)	1478 (34.78%)	407 (39.67%)
Beta blockers	48,570 (25.3%)	994 (27.35%)	4608 (26.09%)	12,655 (25.28%)	12,898 (26.57%)	5277 (28.60%)	1280 (30.12%)	326 (31.77%)
Statins	82,815 (43.14%)	1456 (40.06%)	7479 (42.35%)	21,479 (42.90%)	22,269 (45.87%)	9210 (49.91%)	2103 (49.48%)	481 (46.88%)
Bronchodilators	21,771 (11.34%)	493 (13.56%)	2059 (11.66%)	5843 (11.67%)	5879 (12.11%)	2435 (13.19%)	581 (13.67%)	123 (11.99%)

*ACE* angiotensin converting enzyme, *ARBs* angiotensin receptor blockers, *CCBs* calcium channel blockers, *DHP*, dihydropyridine, *eGFR* estimated glomerular filtration rate, *MRAs* mineralocorticoid receptor antagonist, *NSAIDs*, nonsteroidal anti-inflammatory drugs, *RAASi* renin-angiotensin-aldosterone system inhibitor, *SD* standard deviation ^a^Stratified baseline characteristics include only those patients with an observed serum potassium measurement recorded within + 3 months of the index date ^b^Baseline clinical measurements defined as the first measurement recorded within + 3 months of the index date ^c^Defined as clinical history over five years (60 months) prior to the index date ^d^Defined as medication prescribed within ±3 months of the index date ^e^RAASi therapy was comprised of ACE inhibitors, ARBs, renin inhibitors and MRAs

Approximately half of patients (48.06%) received RAASi therapy, with ACE inhibitors being the most commonly used agent (35.36%). When stratified according to baseline serum potassium, patients with increasing potassium levels were typically more likely to receive ACE inhibitors, ARBs and MRAs. Other frequently prescribed medications included statins (43.14%), diuretics (40.19%), β-blockers (25.30%), and dihydropyridine calcium channel blockers (23.78%).

In the absence of CKD-specific dosing guidelines, Table 2 presents the proportion of patients who received RAASi agents at doses recommended in European Society of Cardiology guidelines for heart failure management [9]. Over one-third of patients receiving ACE inhibitors or MRAs (36.11% and 36.02%, respectively) achieved the target maintenance dose for these therapies; in contrast, 3.28% of those receiving ARBs achieved the target dose. The majority of ARB-treated patients (59.48%) achieved less than 50% of the recommended target dose, compared to 37.38% of patients receiving ACE inhibitors. No patients in receipt of MRAs achieved less than 50% of the recommended target dose.

**Table 2:** Renin-angiotensin-aldosterone system inhibitor use at baseline, relative to target maintenance doses recommended by European Society of Cardiology guidelines for heart failure management [9]

RAASi agent	Number of patients	RAASi dosing at baseline, n (%)^a^		
		≥100% of target	50% to < 100% of target	< 50% of target
ACE inhibitors^b^	53,571	19,345 (36.11%)	14,202 (26.51%)	20,024 (37.38%)
ARBs^c^	18,671	613 (3.28%)	6953 (37.24%)	11,105 (59.48%)
MRAs^d^	3770	1358 (36.02%)	2412 (63.98%)	0 (0.00%)

*ACE* angiotensin converting enzyme, *ARBs* angiotensin receptor blockers, *MRAs* mineralocorticoid receptor antagonist, *RAASi* renin-angiotensin-aldosterone system inhibitors ^a^Defined as medication prescribed within ±3 months of the index date ^b^ACE inhibitors included ramipril, lisinopril, enalapril maleate and captopril ^c^ARBs included candesartan cilexetil, losartan potassium and valsartan ^d^MRAs included spironolactone and eplerenone

### Serum potassium and hyperkalaemia

Using serum potassium intervals of ≥5.0 mmol/L to < 5.5 mmol/L, ≥5.5 mmol/L to < 6.0 mmol/L, and ≥ 6.0 mmol/L to define hyperkalaemia of increasing severity, 86,691 (45.16%), 30,629 (15.96%) and 9440 (4.92%) patients experienced at least one episode over the follow-up period, respectively (Table 3). Based on these serum potassium thresholds, crude incidence rates of hyperkalaemia were 246.02, 62.66 and 15.00 events per 1000 patient-years, respectively. Hyperkalaemia recurrence exhibited a notable pattern; time intervals between successive episodes of serum potassium ≥5.0 mmol/L to < 5.5 mmol/L decreased from 1.80 years (time to first episode), to 0.99 years (first to second episode), to 0.76 years (second to third episode), and to 0.61 years (third to fourth episode). Similar patterns held true when hyperkalaemia was defined according to serum potassium intervals of ≥5.5 mmol/L to < 6.0 mmol/L and ≥ 6.0 mmol/L.

**Table 3:** Incidence and median time between hyperkalaemia episodes

Statistic	Serum potassium interval to define hyperkalaemia^a^		
	≥5.0 mmol/L to < 5.5 mmol/L	≥5.5 mmol/L to < 6.0 mmol/L	≥6.0 mmol/L
Incidence of hyperkalaemia			
Total hyperkalaemia episodes	234,339	59,688	14,291
Mean number of hyperkalaemia episodes per patient (SD)	1.22 (2.29)	0.31 (1.05)	0.07 (0.44)
Crude rate of hyperkalaemia per 1000 patient-years (95% CI)	246.02 (245.03, 247.02)	62.66 (62.16, 63.17)	15.00 (14.76, 15.25)
Number patients experiencing hyperkalaemia episodes (%)			
No hyperkalaemia episodes	105,273 (54.84%)	161,335 (84.04%)	182,524 (95.08%)
Exactly one hyperkalaemia episode	38,323 (19.96%)	18,765 (9.78%)	6985 (3.64%)
Exactly two hyperkalaemia episodes	18,253 (9.51%)	5683 (2.96%)	1449 (0.75%)
Exactly three hyperkalaemia episodes	10,352 (5.39%)	2606 (1.36%)	490 (0.26%)
Four or more hyperkalaemia episodes	19,763 (10.30%)	3575 (1.86%)	516 (0.27%)
To first episode (among patients experiencing 1+ episodes)	1.80 (0.72, 3.61)	2.58 (1.13, 4.62)	3.09 (1.41, 5.17)
First to second episode (among patients experiencing 2+ episodes)	0.99 (0.40, 1.99)	0.84 (0.24, 1.87)	0.65 (0.12, 1.66)
Second to third episode (among patients experiencing 3+ episodes)	0.76 (0.31, 1.48)	0.59 (0.19, 1.31)	0.41 (0.10, 1.13)
Third to fourth episode (among patients experiencing 4+ episodes)	0.61 (0.26, 1.18)	0.48 (0.15, 1.06)	0.30 (0.08, 0.84)

*CI* confidence interval, *IQR* inter-quartile range, *SD* standard deviation ^a^Hyperkalaemia episodes of increasing severity were quantified as serum potassium ≥5.0 mmol/L to < 5.5 mmol/L, ≥5.5 mmol/L to < 6.0 mmol/L, and ≥ 6.0 mmol/L, without a measurement of at least this severity (≥5.0 mmol/L, ≥5.5 mmol/L, and ≥ 6.0 mmol/L, respectively) in the preceding seven days

### Incidence of death, MACE, and RAASi discontinuation

In total, 44,961 deaths, 80,038 MACE and 75,488 RAASi discontinuation events were observed over the follow-up period, which corresponded to crude event rates of 47, 84 and 79 per 1000 patient-years, respectively (Additional file 1: Table S2).

**Fig. 2:**
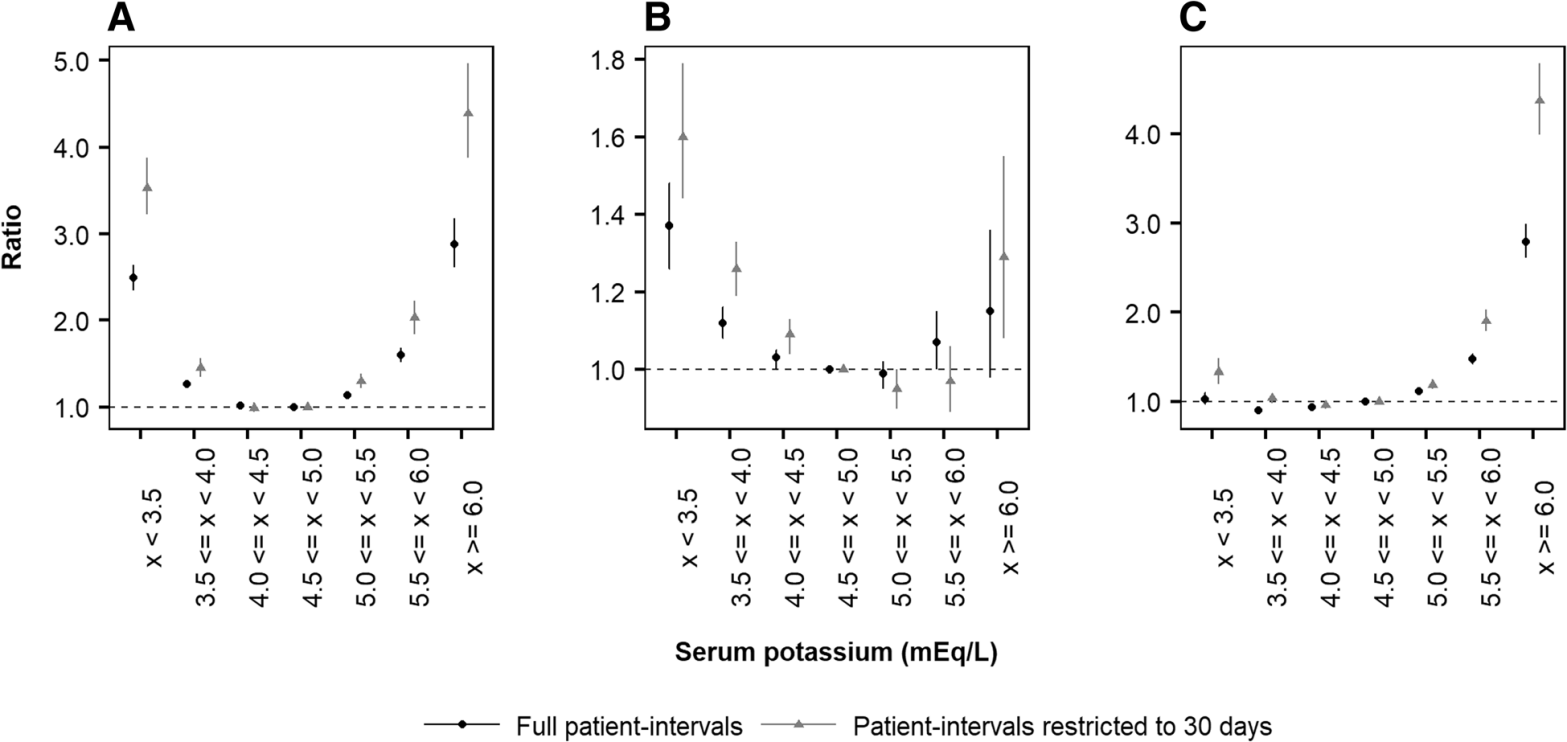
Adjusted incident rate ratios for death (**a**), MACE (**b**) and RAASi discontinuation (**c**) as a function of serum potassium in CKD patients**.** *Black: full patient-intervals; grey: patient-intervals restricted to 30 days. Incident rate ratios were adjusted to account for confounding patient demographics, clinical histories and comorbidities, clinical measurements, and medication usage, as reported in Table **4 **and Additional file **1**: Table S3. CKD: chronic kidney disease; MACE: major adverse cardiac event; RAASi: renin-angiotensin-aldosterone system inhibitor. Error bars represent 95% confidence intervals*

When considering the full duration of patient-intervals between serum potassium measurements, adjusted IRRs for death exhibited a U-shaped association with categorical serum potassium levels (Fig. 2a). Relative to the reference category of 4.5 to < 5.0 mmol/L, serum potassium < 4.0 mmol/L and ≥ 5.0 mmol/L were each associated with increased mortality risk. Among patients with serum potassium ≥5.5 mmol/L to < 6.0 mmol/L, the adjusted IRR for mortality was 1.60 (95% CI: 1.52–1.68) compared with the reference category, which increased to 2.88 (2.61–3.18) in patients with serum potassium ≥6.0 mmol/L. In validation, the U-shaped association pattern between potassium and mortality observed in this study was similar to that published by Luo et al. [4] (Additional file 3: Figure S2); in the case of the latter, an adjusted IRR of 1.60 (1.37–1.88) was estimated for patients with serum potassium ≥5.5 mmol/L to < 6.0 mmol/L (as observed in our study), but a somewhat larger IRR of 3.31 (2.52–4.34) was estimated for patients with serum potassium ≥6.0 mmol/L (compared to 2.88 in our study).

Similar to mortality, serum potassium levels < 4.0 mmol/L were associated with increased risks of MACE when the full duration of patient-intervals were utilised (Fig. 2b). However, confidence intervals relating to serum potassium ≥5.0 mmol/L contained an IRR of unity, reflecting increased uncertainty of the association between hyperkalaemia and MACE. Relative to the reference category, adjusted IRRs for MACE were 1.07 (1.00–1.15) and 1.15 (0.98–1.36) for patients with serum potassium ≥5.5 mmol/L to < 6.0 mmol/L and ≥ 6.0 mmol/L, respectively. In validation, the association between serum potassium and MACE estimated by Luo et al. [4] exhibited a more pronounced U-shaped pattern than that observed in our study (Additional file 3: Figure S2), with larger and statistically significant adjusted IRRs of 1.12 (1.05–1.20) and 1.88 (1.66–2.12) for patients with serum potassium ≥5.5 mmol/L to < 6.0 mmol/L and ≥ 6.0 mmol/L, respectively.

Associations between adverse event risk and serum potassium concentration were more pronounced after restricting patient-intervals to a maximum of 30 days from each serum potassium measurement. Relative to the reference category, adjusted IRRs for patients with serum potassium ≥5.5 mmol/L to < 6.0 mmol/L were estimated as 2.03 (1.84–2.23) and 0.97 (0.89–1.06) for mortality and MACE, respectively; these increased to 4.39 (3.88–4.97) and 1.29 (1.08–1.55), respectively, among patients with serum potassium ≥6.0 mmol/L.

The association between categorical serum potassium and the incidence of RAASi discontinuation was observed to be J-shaped (Fig. 2c). Based on the full duration of patient-intervals between serum potassium measurements, adjusted IRRs for RAASi discontinuation were estimated as 1.47 (1.42–1.53) for patients with serum potassium ≥5.5 mmol/L to < 6.0 mmol/L, and increased to 2.79 (2.61–2.99) among those with serum potassium ≥6.0 mmol/L, when compared to the reference category. Following restriction of patient-intervals to a maximum of 30 days from each serum potassium measurement, adjusted IRRs for RAASi discontinuation increased to 1.90 (1.79–2.03) and 4.37 (3.99–4.79) among patients with serum potassium ≥5.5 mmol/L to < 6.0 mmol/L and ≥ 6.0 mmol/L, respectively.

Observed associations between serum potassium and clinical outcomes were preserved when patients were stratified by CKD stage in sensitivity analyses (Additional file 4: Figure S3). Adjusted IRRs for mortality, MACE and RAASi discontinuation in patients with CKD 3a, CKD 3b, CKD 4 and CKD 5 demonstrated similar trends to one another and to the overall cohort; however, smaller sample sizes gave rise to wider confidence intervals and greater uncertainty, particularly for patients with eGFR < 15 mL/min/1.73m^2^.

Table 4 summarises coefficient estimates and statistical inferences relating to the final fitted risk equations. In addition to serum potassium concentration, age and sex were significant risk factors for both death and MACE (*P* < 0.0001). Death was also significantly associated with RAASi usage, time with CKD and eGFR, while history of MACE of baseline was a significant risk factor for MACE during follow-up (all *P* < 0.0001). For RAASi discontinuation, the most important risk factors were eGFR, diuretics usage, diabetes status and sex (all *P* < 0.0001). Coefficient estimates and statistical inferences obtained after re-estimating the equations on patient-intervals restricted to a maximum of 30 days are provided in Additional file 1 (Table S3).

**Table 4:** Model output for final risk equations

Explanatory variable	Estimate	SE	*t* statistic	*P*-value
Incidence of death				
Constant	−4.1733	0.2272	−18.37	< 0.0001
Serum potassium: < 3.5 mmol/L	0.9137	0.0300	30.47	< 0.0001
Serum potassium: 3.5 to < 4.0 mmol/L	0.2385	0.0171	13.96	< 0.0001
Serum potassium: 4.0 to < 4.5 mmol/L	0.0184	0.0127	1.45	0.1395
Serum potassium: 5.0 to < 5.5 mmol/L	0.1304	0.0159	8.20	< 0.0001
Serum potassium: 5.5 to < 6.0 mmol/L	0.4689	0.0262	17.91	< 0.0001
Serum potassium: ≥6.0 mmol/L	1.0578	0.0499	21.20	< 0.0001
Age at baseline (years)	0.0715	0.0008	95.05	< 0.0001
Sex at baseline: Female	−0.3021	0.0111	−27.18	< 0.0001
Smoker at baseline: Yes	0.4211	0.0157	26.90	< 0.0001
Time with CKD (years)	0.0994	0.0022	44.71	< 0.0001
Time-updated eGFR (mL/min/1.73m^2^; truncated at 60 mL/min/1.73m^2^)	− 0.0240	0.0006	−43.32	< 0.0001
Time-updated prescribed RAASi: Yes	−0.7887	0.0113	−69.54	< 0.0001
Time-updated history of HF: Yes	0.8336	0.0213	39.08	< 0.0001
History of diabetes at baseline: Yes	0.3289	0.0160	20.58	< 0.0001
History of cancer at baseline: Yes	0.3858	0.0171	22.60	< 0.0001
History of PVD at baseline: Yes	0.2370	0.0290	8.17	< 0.0001
History of dementia at baseline: Yes	0.7182	0.0251	28.67	< 0.0001
History of MACE at baseline: Yes	0.2946	0.0133	22.12	< 0.0001
Natural logarithm of baseline BMI (kg/m^2^)	−0.9316	0.0601	−15.51	< 0.0001
Natural logarithm of baseline haemoglobin (g/cL)	−0.8928	0.0392	−22.76	< 0.0001
Prescribed diuretics ±3 months of baseline: Yes	0.1574	0.0119	13.28	< 0.0001
Prescribed bronchodilators ±3 months of baseline: Yes	0.3514	0.0154	22.81	< 0.0001
Prescribed insulin ±3 months of baseline: Yes	0.3265	0.0338	9.66	< 0.0001
Prescribed statins ±3 months of baseline: Yes	−0.1796	0.0116	−15.49	< 0.0001
Incidence of MACE				
Constant	−5.7072	0.0967	−59.01	< 0.0001
Serum potassium: < 3.5 mmol/L	0.3126	0.0416	7.52	< 0.0001
Serum potassium: 3.5 to < 4.0 mmol/L	0.1117	0.0199	5.62	< 0.0001
Serum potassium: 4.0 to < 4.5 mmol/L	0.0257	0.0140	1.84	0.0731
Serum potassium: 5.0 to < 5.5 mmol/L	−0.0110	0.0182	− 0.61	0.3322
Serum potassium: 5.5 to < 6.0 mmol/L	0.0722	0.0353	2.05	0.0492
Serum potassium: ≥6.0 mmol/L	0.1426	0.0825	1.73	0.0897
Age at baseline (years)	0.0407	0.0007	55.07	< 0.0001
Sex at baseline: Female	−0.2540	0.0148	−17.18	< 0.0001
Smoker at baseline: Yes	0.0628	0.0198	3.17	0.0027
Time with CKD (years)	0.0714	0.0027	26.33	< 0.0001
Time-updated eGFR (mL/min/1.73m^2^)	−0.0032	0.0007	−4.88	< 0.0001
History of diabetes at baseline: Yes	0.0518	0.0208	2.49	0.0181
History of MACE at baseline: Yes	0.7804	0.0174	44.80	< 0.0001
History of rheumatologic disease at baseline: Yes	0.0930	0.0364	2.55	0.0153
History of CPD at baseline: Yes	0.2485	0.0220	11.29	< 0.0001
Natural logarithm of baseline total cholesterol (mmol/L)	−0.1001	0.0345	−2.91	0.0066
Prescribed CCBs ±3 months of baseline: Yes	0.1042	0.0152	6.86	< 0.0001
Prescribed insulin ±3 months of baseline: Yes	0.2142	0.0416	5.14	< 0.0001
Prescribed beta blockers ±3 months of baseline: Yes	0.2504	0.0156	16.06	< 0.0001
Incidence of RAASi discontinuation				
Constant	−1.6740	0.0254	−65.84	< 0.0001
Serum potassium: < 3.5 mmol/L	0.0316	0.0329	0.96	0.2519
Serum potassium: 3.5 to < 4.0 mmol/L	−0.1005	0.0144	−6.96	< 0.0001
Serum potassium: 4.0 to < 4.5 mmol/L	−0.0653	0.0095	−6.87	< 0.0001
Serum potassium: 5.0 to < 5.5 mmol/L	0.1148	0.0115	10.00	< 0.0001
Serum potassium: 5.5 to < 6.0 mmol/L	0.3858	0.0192	20.09	< 0.0001
Serum potassium: ≥6.0 mmol/L	1.0271	0.0352	29.16	< 0.0001
Sex at baseline: Female	−0.1364	0.0094	−14.45	< 0.0001
Time with CKD (years)	0.0161	0.0017	9.44	< 0.0001
Time-updated eGFR (mL/min/1.73m^2^; truncated at 60 mL/min/1.73m^2^)	−0.0205	0.0004	−45.54	< 0.0001
History of diabetes at baseline: Yes	0.2476	0.0128	19.32	< 0.0001
History of rheumatologic disease at baseline: Yes	0.1235	0.0227	5.45	< 0.0001
History of MACE at baseline: Yes	0.1432	0.0112	12.76	< 0.0001
Prescribed diuretics ±3 months of baseline: Yes	0.2374	0.0096	24.60	< 0.0001
Prescribed insulin ±3 months of baseline: Yes	0.1519	0.0239	6.34	< 0.0001
Prescribed CCBs ±3 months of baseline: Yes	0.1197	0.0099	12.14	< 0.0001

*BMI* body mass index, *CCB* calcium-channel blocker, *CKD* chronic kidney disease, *CPD* chronic pulmonary disease, *eGFR* estimated glomerular filtration rate, *HF* heart failure, *MACE* major adverse cardiac events, *PVD* peripheral vascular disease, *RAASi* renin-angiotensin-aldosterone system inhibitor, *SE* standard error

**Fig. 3:**
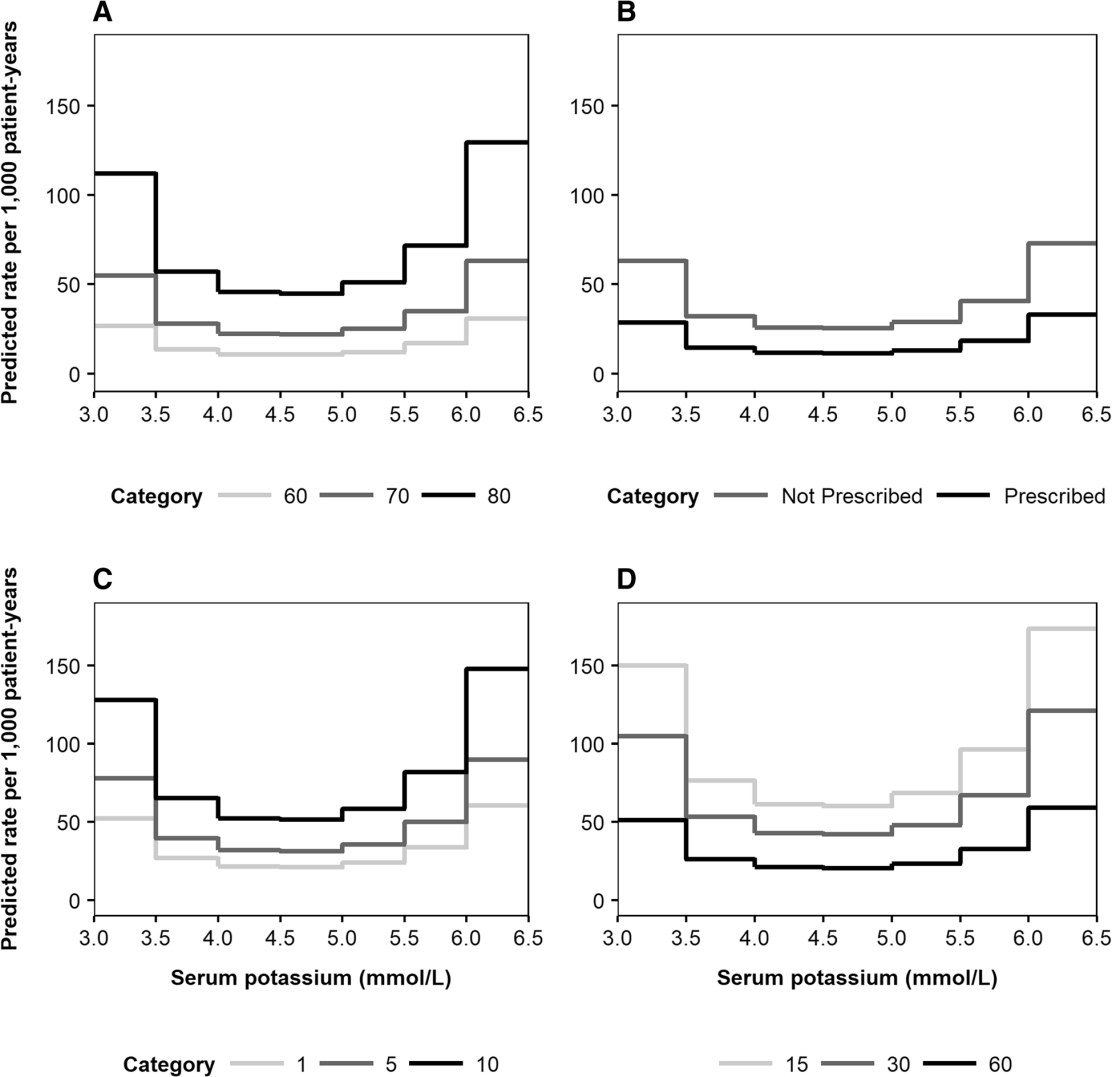
Predicted incidence rates of death, disaggregated by age (**a**), RAASi usage (**b**), time from index (**c**) and eGFR (**d**)***. a*** *light grey line: 60 years, dark grey line: 70 years, black line: 80 years.* **b** *grey line: not prescribed, black line: prescribed.* **c** *light grey line: 1 year, dark grey line: 5 years, black line: 10 years.* **d** *light grey line: 15 mL/min/1.73m*^*2*^*, dark grey line: 30 mL/min/1.73m*^*2*^*, black line: 60 mL/min/1.73m*^*2*^*. The four most important variables for each event (according to the absolute value of the t statistic) were varied, with all other baseline covariates reflective of the cohort average: female; aged 72 years; non-smoker; no history of comorbidities; no medications prescribed; 1066 days elapsed since initial CKD event; no history of heart failure during follow-up period; eGFR 51 mL/min/1.73m*^*2*^*; BMI 29 kg/m*^*2*^*; haemoglobin 13.6 g/dL; total cholesterol 4.98 mmol/L. BMI: body mass index; CKD: chronic kidney disease; eGFR: estimated glomerular filtration rate; RAASi: renin-angiotensin-aldosterone system inhibitor*

**Fig. 4:**
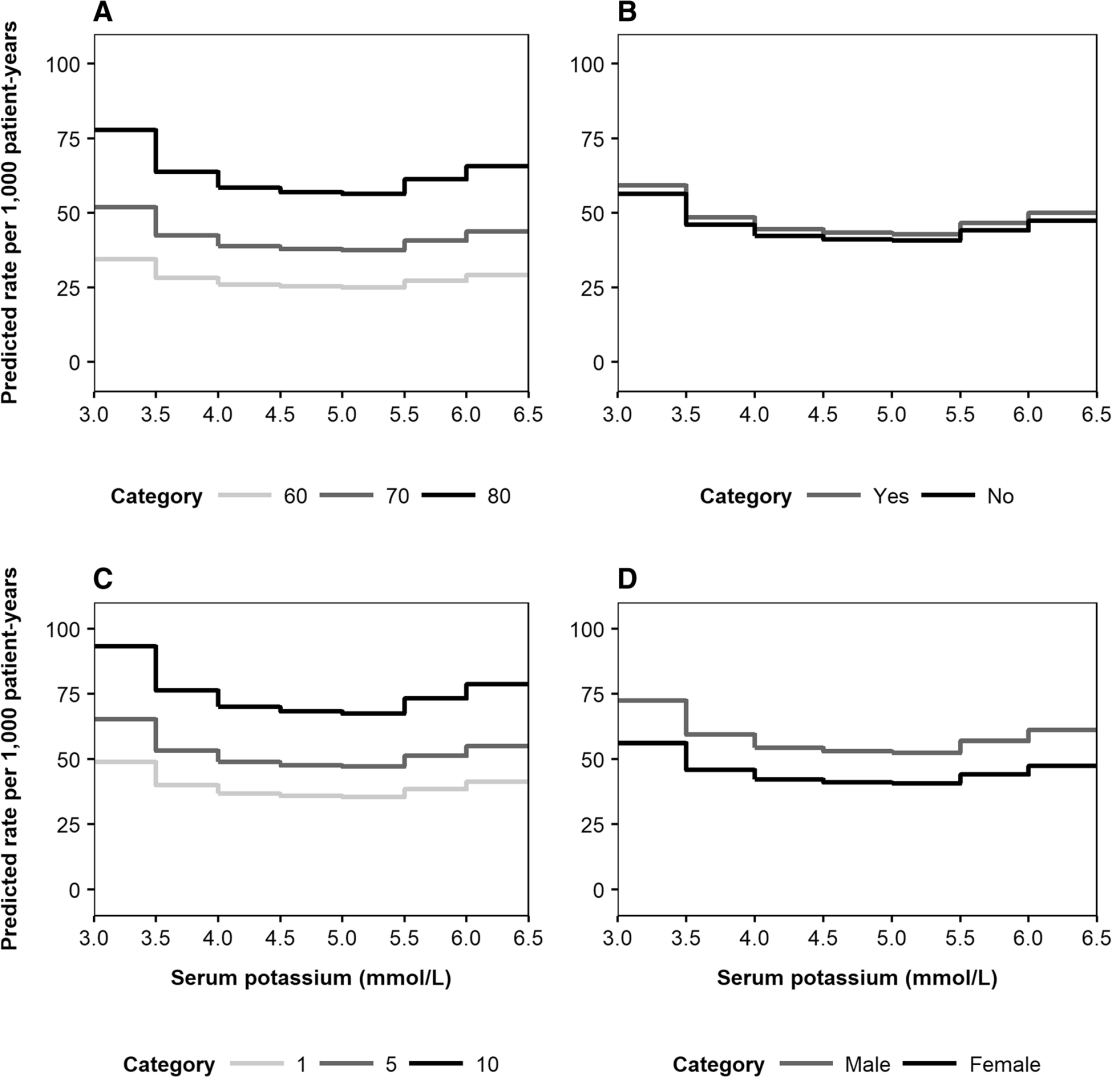
Predicted incidence rates of MACE, disaggregated by age (**a**), history of MACE (**b**), time from index (**c**) and sex (**d**)***. a*** *light grey line: 60 years, dark grey line: 70 years, black line: 80 years.* **b** *grey line: yes, black line: no.* **c** *light grey line: 1 year, dark grey line: 5 years, black line: 10 years.* **d** *grey line: male, black line: female. The four most important variables for each event (according to the absolute value of the t statistic) were varied, with all other baseline covariates reflective of the cohort average: female; aged 72 years; non-smoker; no history of comorbidities; no medications prescribed; 1066 days elapsed since initial CKD event; no history of heart failure during follow-up period; eGFR 51 mL/min/1.73m*^*2*^*; BMI 29 kg/m*^*2*^*; haemoglobin 13.6 g/dL; total cholesterol 4.98 mmol/L. BMI: body mass index; CKD: chronic kidney disease; eGFR: estimated glomerular filtration rate; MACE: major adverse cardiac event*

**Fig. 5:**
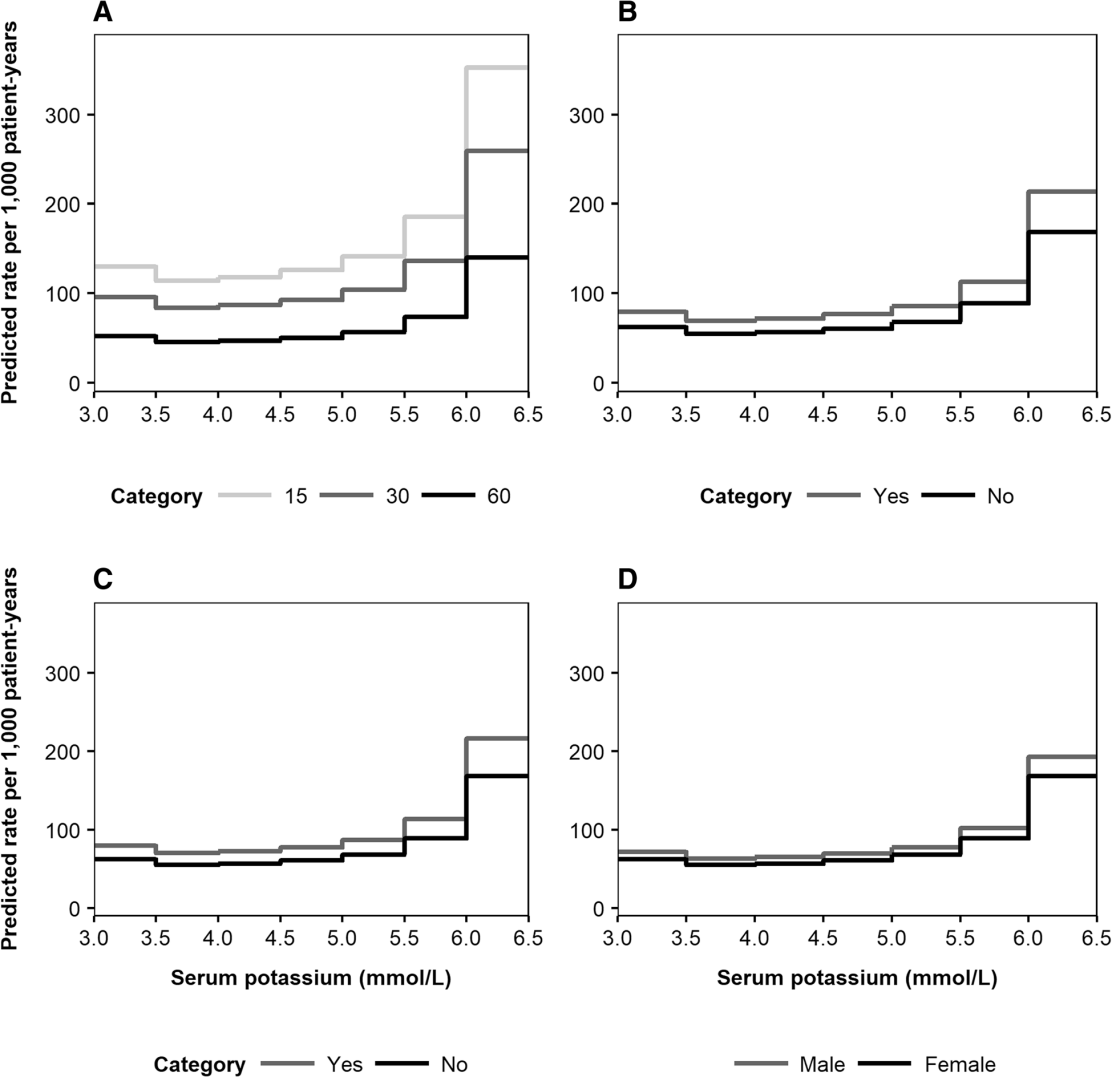
Predicted incidence rates of RAASi discontinuation, disaggregated by eGFR (**a**), diuretics usage (**b**), presence of diabetes (**c**), and sex (**d**)***. a*** *light grey line: 15 mL/min/1.73m*^*2*^*, dark grey line: 30 mL/min/1.73m*^*2*^*, black line: 60 mL/min/1.73m*^*2*^*.* **b** *grey line: yes, black line: no.* **c** *grey line: yes, black line: no.* **d** *grey line: male, black line: female. The four most important variables for each event (according to the absolute value of the t statistic) were varied, with all other baseline covariates reflective of the cohort average: female; aged 72 years; non-smoker; no history of comorbidities; no medications prescribed; 1066 days elapsed since initial CKD event; no history of heart failure during follow-up period; eGFR 51 mL/min/1.73m*^*2*^*; BMI 29 kg/m*^*2*^*; haemoglobin 13.6 g/dL; total cholesterol 4.98 mmol/L. BMI: body mass index; CKD: chronic kidney disease; eGFR: estimated glomerular filtration rate; RAASi: renin-angiotensin-aldosterone system inhibitor*

To illustrate the predictive outputs of the risk equations, Fig. 3 presents the expected rates of death (per 1000 patient-years) as a function of serum potassium level. Predicted mortality rates were further disaggregated by the four most important predictive factors: age, RAASi usage, time with CKD and eGFR (Fig. 3a, b, c and d, respectively). Similarly, association patterns between serum potassium and MACE incidence, disaggregated by age, history of MACE, time from index and sex, are illustrated in Fig. 4a, b, c and d, respectively. Expected rates of RAASi discontinuation according to serum potassium level are presented in Fig. 5, disaggregated by eGFR, diuretics usage, diabetes status and sex (Fig. 5a, b, c and d, respectively).

## Discussion

Using real-world data from 191,964 CKD patients in the UK, we derived risk equations that may be used in clinical practice to relate serum potassium and other predictive factors to the incidence of mortality, MACE and RAASi discontinuation. Adjusted IRRs derived in this analysis demonstrated U-shaped associations between serum potassium and the risks of mortality and MACE (though not as strongly in the case of the latter); and a J-shaped association between serum potassium and the incidence of RAASi discontinuation. Other statistically important predictors of adverse clinical outcomes included age and sex, time with CKD, RAASi usage, eGFR, and history of MACE. The risk equations presented here represent a valuable tool to identify CKD patients at increased risk of adverse clinical outcomes; and those most likely to benefit from strategies that avoid hyperkalaemia, prevent RAASi discontinuation, and effectively maintain serum potassium homeostasis.

Our findings are broadly consistent with literature reporting relationships between hypo- and hyperkalaemia and worsening clinical outcomes in CKD patients [2–5]. In particular, mortality risk was lowest among patients with serum potassium between 4.0–5.0 mmol/L; an optimal range similarly identified in recent US studies associating serum potassium with all-cause mortality in patients with and without CKD, HF and/or diabetes [6], and those examining in-hospital mortality risk among hospitalised patients with CKD and/or cardiovascular disease [21]. Other US studies have also demonstrated significant relationships between all-cause mortality and both hypo- (< 3.5 mmol/L) and hyperkalaemia (> 5.0 mmol/L) in patients with CKD stages 3–4 [22]; and between both all-cause mortality and hospital admissions and hyperkalaemia (> 5.0 mmol/L) in cardiovascular disease patients treated with antihypertensive medications that impair potassium homeostasis [23]. Although cause of death was not explored in our analysis due to a paucity of relevant CPRD data, a previous study of CKD patients with coronary artery disease found that the risk of sudden cardiac arrest and death doubled when serum potassium measurements taken before the event exceeded 5.0 mmol/L [24].

Additional sensitivity analyses found that observed relationships between serum potassium and mortality, MACE and RAASi discontinuation were preserved when patients were stratified by CKD stage, with little variation in the association patterns between stages. This finding is consistent with a similar study by Luo et al., who found no significant interaction between eGFR and serum potassium when predicting mortality in a US CKD 3+ population [4]. However, it is noteworthy that observed relationships between serum potassium and risks of mortality and MACE in the overall CKD cohort were comparatively weaker than those estimated by Luo et al. [4]. While our study examined an incident CKD cohort, associations reportered herein may have been strengthened by the inclusion of a prevalent CKD population. Death within 30 days of MACE occurred for approximately 2% of MACE events recorded in this study; therefore, relationships between serum potassium and MACE may be underestimated, due to potential under-reporting of fatal MACE in primary care and a lack of cause of death data. Moreover, although previous studies have generally found that deaths recorded on the CPRD are in concordance with Office for National Statistics national mortality registrations [25], less is known about the reverse. However, an important consideration is the difference in contextual settings and populations captured by each database, particularly regarding patient demographics, clinical characteristics, treatment paradigms and healthcare systems. One notable strength of our study is the use of the CPRD, which contains primary care data for a large, diverse population with access to publicly-funded healthcare. Compared to existing US data, it could therefore be suggested that our results are more generalisable to other countries and healthcare systems, particularly those across Europe.

We demonstrated a J-shaped association pattern between serum potassium and RAASi discontinuation risk. Adjusted IRRs reported here relate to RAASi discontinuation rates estimated among all patients and intervals observed during the study period. However, when alternative definitions of RAASi exposure were investigated (such as only including patients in receipt of RAASi over the duration of the follow-up period, or intervals where a RAASi therapy had been prescribed), the association pattern between serum potassium levels and the likelihood of RAASi discontinuation was preserved. This J-shaped association is consistent with prescribing guidelines, where hyperkalaemia risk among patients treated with RAASi is commonly managed through the down-titration or discontinuation of such potassium-retaining agents [9, 10]. When delivered at guideline-recommended doses, RAASi have been evidenced to control blood pressure, delay disease progression and improve long-term outcomes in CKD patients [7, 9, 26–28]; however, suboptimal dosing has conversely been associated with increased adverse event risks and healthcare costs [11–14]. Consequently, strategies to control serum potassium and avoid RAASi discontinuation represent an important advance in CKD management, and have significant potential to improve health economic outcomes. The risk equations generated in this study are intended to inform the potential value of maintaining normal serum potassium levels in CKD patients, and identify clinical factors that place patients at greater risk of RAASi discontinuation, MACE and death.

There is no single definition of hyperkalaemia; therefore, comparisons of incidence and outcomes across epidemiological studies are often obscured by inconsistent serum potassium thresholds. As shown in this study, the incidence of hyperkalaemia was highly sensitive to the serum potassium threshold used to define it; and estimated IRRs of death, MACE and RAASi discontinuation were higher with increasing serum potassium levels. Had the sample size allowed for such analysis, we believe that further stratification of patients with serum potassium ≥6.0 mmol/L would have strengthened the association pattern between serum potassium and adverse event risk. Nevertheless, our results highlight the importance of a standardised definition of hyperkalaemia, to facilitate valid comparisons between observational studies and across different populations.

To our knowledge, few studies have examined the time between successive hyperkalaemia episodes in patients with recurrent hyperkalaemia. A noteworthy pattern observed in our analyses was the shortening of intervals between successive hyperkalaemia episodes, irrespective of the serum potassium intervals used to define events of increasing severity (≥5.0 mmol/L to < 5.5 mmol/L, ≥5.5 mmol/L to < 6.0 mmol/L, and ≥ 6.0 mmol/L). Given the impact of hyperkalaemia on patient morbidity, mortality and associated resource utilisation, our findings highlight the importance of sustained potassium management following discharge from immediate medical care, in order to avoid the burden of recurrent hyperkalaemia on patients and healthcare systems.

A number of limitations of this study are worth noting, and predominantly relate to the constraints of the data used to inform our analyses. The CPRD dataset may overestimate the incidence of hyperkalaemia due to pseudo-hyperkalaemia, a consequence of haemolysis following traumatic venepuncture, fist clenching during phlebotomy, blood sample contamination, inadequate blood sample storage, and delays between venepuncture and sample analysis [29]. Although causality cannot be inferred from this retrospective, observational database study, estimated IRRs for death, MACE and RAASi discontinuation were adjusted to account for confounding patient demographics, clinical histories and comorbidities, clinical measurements, and selected medication usage. Declining renal function was controlled for in our risk equations by including eGFR as a time-varying covariate; however, we acknowledge that related indices of kidney function (such as proteinuria) were not included in our analyses. Other candidate covariates were limited to those variables available within the CPRD; consequently, a number of unobserved or poorly-recorded potential confounders were not considered, such as socio-demographics, diet, lifestyle, ethnicity, smoking status, and the use of potassium-binding agents. Despite these limitations, the size of the CPRD dataset allowed this study to produce statistically-robust estimates of adverse event risk that verified the association curves observed across different geographical locations and comorbid populations.

## Conclusions

In conclusion, this retrospective, observational cohort study sought to describe relationships between serum potassium levels and adverse clinical outcomes among CKD patients in UK clinical practice. Data arising from our analyses emphasise the importance of maintaining serum potassium levels within a narrow physiological range, in order to lower the risks of mortality, MACE and RAASi discontinuation. The risk equations derived represent a valuable tool to predict the incidence of adverse outcomes based on clinical characteristics; and identify CKD patients most likely to benefit from strategies that avoid hypo- and hyperkalaemia, enable optimal RAASi therapy, prevent RAASi discontinuation, and improve long-term outcomes.

## Additional files

Additional file 1: **Table S1.** Read and International Classification of Diseases (ICD-10) codes used to define heart failure and chronic kidney disease. Example of the time-updating methodology used during data structuring. **Table S2.** Incidence of death, major adverse cardiac event and renin-angiotensin-aldosterone system inhibitor discontinuation observed in chronic kidney disease patients, stratified by serum potassium category. **Table S3.** Model output for final risk equations, re-estimated using patient-intervals restricted to a maximum duration of 30 days. (DOCX 58 kb)

Additional file 2: **Figure S1.** Illustrative example of time-updated patient-intervals based on timing of serum potassium measurements. eGFR: estimated glomerular filtration rate; K^+^: serum potassium; MACE: major adverse cardiac event. (TIF 260 kb)

Additional file 3: **Figure S2.** Validation of adjusted incident rate ratios for death (A) and MACE (B) as a function of serum potassium against those published by Luo et al. Black: estimated in the present study (UK CKD stage 3+ patients); grey: estimated by Luo et al. (US CKD stage 3+ patients). Error bars represent 95% confidence intervals. (TIFF 41 kb)

Additional file 4: **Figure S3.** Adjusted incident rate ratios for death (A), MACE (B) and RAASi discontinuation (C) as a function of serum potassium in patients stratified by CKD stage. Black: CKD 3a patients; dark grey: CKD 3b patients; mid-grey: CKD 4 patients; light grey: CKD 5 patients. Incident rate ratios were adjusted to account for confounding patient demographics, clinical histories and comorbidities, clinical measurements, and medication usage, as reported in Table 4. CKD: chronic kidney disease; MACE: major adverse cardiac event; RAASi: renin-angiotensin-aldosterone system inhibitor. Error bars represent 95% confidence intervals. (TIFF 63 kb)

## Abbreviations

ACE: Angiotensin-converting enzyme
ARB: Angiotensin II receptor blocker
CI: Confidence interval
CKD: Chronic kidney disease
CPRD: Clinical Practice Research Datalink
eGFR: Estimated glomerular filtration rate
HES: Hospital Episode Statistics
HF: Heart failure
IRR: Incident rate ratio
LOCF: Last-observation-carried-forward
MACE: Major adverse cardiac event
MRA: Mineralocorticoid receptor antagonist
RAASi: Renin-angiotensin-aldosterone system inhibitor
