# Supplementary material for: Serum potassium as a predictor of adverse clinical outcomes in patients with chronic kidney disease: new risk equations using the UK clinical practice research datalink
Source: BMC Nephrol. 2018 Aug 22;19:211. doi: 10.1186/s12882-018-1007-1 (PMC6106824; doi:10.1186/s12882-018-1007-1)
Supplement: Supplementary file 1 — Table S1. Read and International Classification of Diseases (ICD-10) codes used to define heart failure and chronic kidney disease. Example of the time-updating methodology used during data structuring. Table S2. Incidence of death, major adverse cardiac event and renin-angiotensin-aldosterone system inhibitor discontinuation observed in chronic kidney disease patients, stratified by serum potassium category. Table S3. Model output for final risk equations, re-estimated using patient-intervals restricted to a maximum duration of 30 days. (DOCX 58 kb) [file 12882_2018_1007_MOESM1_ESM.docx]

# Additional File 1

**Table S1** Read and International Classification of Diseases (ICD-10) codes used to define heart failure and chronic kidney disease

| **Description** | **Code** |
| --- | --- |
| **Heart failure – Read codes** | |
| Congestive heart failure | 4270 |
| LVF (left ventricular failure) | 4271 |
| H/O: Heart failure | 14A6.00 |
| H/O: Heart failure in last year | 14AM.00 |
| Heart failure confirmed | 1O1..00 |
| Hypertension congestive heart failure | 402 C |
| Congestive cardiac failure | 4270C |
| Congestive heart failure compensated | 4270CC |
| Congestive heart failure decompensated | 4270D |
| Heart failure right-sided | 4270R |
| Left ventricular failure acute | 4271A |
| Heart failure left-sided | 4271H |
| Heart failure annual review | 662W.00 |
| Heart failure acute | 7824AC |
| Cardiac failure therapy | 8B29.00 |
| Heart failure care plan discussed with patient | 8CL3.00 |
| Admit heart failure emergency | 8H2S.00 |
| Hypertensive heart & renal dysfunction with (congestive) heart failure | G232.00 |
| Heart failure | G58..00 |
| Cardiac failure | G58..11 |
| Congestive heart failure | G580.00 |
| Congestive cardiac failure | G580.11 |
| Right heart failure | G580.12 |
| Acute congestive heart failure | G580000 |
| Chronic congestive heart failure | G580100 |
| Decompensated cardiac failure | G580200 |
| Compensated cardiac failure | G580300 |
| Left ventricular failure | G581.00 |
| Acute left ventricular failure | G581000 |
| Acute heart failure | G582.00 |
| Heart failure NOS | G58z.00 |
| Cardiac failure NOS | G58z.12 |
| Heart failure as a complication of care | SP11111 |
| Right ventricular failure | G580.13 |
| Biventricular failure | G580.14 |
| Right ventricle failure | G584 |
| High output failure (cardiac) | 7824A |
| Failure cardiac | 7824FC |
| Heart failure | 7824FH |
| **Heart failure – ICD-10 codes** | |
| Rheumatic heart failure | I09.81 |
| Rheumatic heart disease, unspecified | I09.9 |
| Hypertensive heart disease with heart failure | I11.0 |
| Hypertensive heart and chronic kidney disease with heart failure and stage 1 through stage 4 chronic kidney disease, or unspecified chronic kidney disease | I13.0 |
| Hypertensive heart and chronic kidney disease with heart failure and stage 1 through stage 4 chronic kidney disease, or unspecified chronic kidney disease | I13.2 |
| Ischaemic cardiomyopathy | I25.5 |
| Dilated cardiomyopathy | I42.0 |
| Other restrictive cardiomyopathy | I42.5 |
| Cardiomyopathy, unspecified | I42.9 |
| Cardiomyopathy in diseases classified elsewhere | I43.X |
| Left ventricular failure | I50.1 |
| Unspecified systolic (congestive) heart failure | I50.20 |
| Acute systolic (congestive) heart failure | I50.21 |
| Chronic systolic (congestive) heart failure | I50.22 |
| Acute on chronic systolic (congestive) heart failure | I50.23 |
| Unspecified diastolic (congestive) heart failure | I50.30 |
| Acute diastolic (congestive) heart failure | I50.31 |
| Chronic diastolic (congestive) heart failure | I50.32 |
| Acute on chronic diastolic (congestive) heart failure | I50.33 |
| Unspecified combined systolic (congestive) and diastolic (congestive) heart failure | I50.40 |
| Acute combined systolic (congestive) and diastolic (congestive) heart failure | I50.41 |
| Chronic combined systolic (congestive) and diastolic (congestive) heart failure | I50.42 |
| Acute on chronic combined systolic (congestive) and diastolic (congestive) heart failure | I50.43 |
| Heart failure, unspecified | I50.9 |
| Postprocedural heart failure following other surgery | I97.131 |
| Postprocedural heart failure | I97.13X |
| Heart failure | l50 |
| Neonatal cardiac failure | P29.0 |
| **Chronic kidney disease – Read codes** | |
| Chronic renal impairment | 1Z1..00 |
| Chronic kidney disease stage 3 | 1Z12.00 |
| Chronic kidney disease stage 4 | 1Z13.00 |
| Chronic kidney disease stage 5 | 1Z14.00 |
| Chronic kidney disease stage 3A | 1Z15.00 |
| Chronic kidney disease stage 3B | 1Z16.00 |
| Chronic kidney disease stage 3 with proteinuria | 1Z1B.00 |
| CKD stage 3 with proteinuria | 1Z1B.11 |
| Chronic kidney disease stage 3 without proteinuria | 1Z1C.00 |
| CKD stage 3 without proteinuria | 1Z1C.11 |
| Chronic kidney disease stage 3A with proteinuria | 1Z1D.00 |
| CKD stage 3A with proteinuria | 1Z1D.11 |
| Chronic kidney disease stage 3A without proteinuria | 1Z1E.00 |
| CKD stage 3A without proteinuria | 1Z1E.11 |
| Chronic kidney disease stage 3B with proteinuria | 1Z1F.00 |
| CKD stage 3B with proteinuria | 1Z1F.11 |
| Chronic kidney disease stage 3B without proteinuria | 1Z1G.00 |
| CKD stage 3B without proteinuria | 1Z1G.11 |
| Chronic kidney disease stage 4 with proteinuria | 1Z1H.00 |
| CKD stage 4 with proteinuria | 1Z1H.11 |
| Chronic kidney disease stage 4 without proteinuria | 1Z1J.00 |
| CKD stage 4 without proteinuria | 1Z1J.11 |
| Chronic kidney disease stage 5 with proteinuria | 1Z1K.00 |
| CKD stage 5 with proteinuria | 1Z1K.11 |
| Chronic kidney disease stage 5 without proteinuria | 1Z1L.00 |
| CKD stage 5 without proteinuria | 1Z1L.11 |
| Compensation for renal failure | 7L1A.00 |
| Renal failure-associated hyperphosphataemia | C353600 |
| Anaemia secondary to renal failure | D215.00 |
| Anaemia secondary to chronic renal failure | D215000 |
| Hypertensive renal disease with renal failure | G222.00 |
| Hypertensive heart and renal disease with renal failure | G233.00 |
| Acute renal failure | K04..00 |
| ARF - Acute renal failure | K04..11 |
| Other acute renal failure | K04y.00 |
| Acute renal failure NOS | K04z.00 |
| Chronic renal failure | K05..00 |
| End stage renal failure | K05..12 |
| Chronic kidney disease | K05..13 |
| End stage renal failure | K050.00 |
| Chronic kidney disease stage 3 | K053.00 |
| Chronic kidney disease stage 4 | K054.00 |
| Chronic kidney disease stage 5 | K055.00 |
| Renal failure unspecified | K06..00 |
| Kidney failure unspecified | K06..12 |
| End-stage renal disease | K0D..00 |
| Acute-on-chronic renal failure | K0E..00 |
| Renal failure | Kyu2.00 |
| Other acute renal failure | Kyu2000 |
| Other chronic renal failure | Kyu2100 |
| **Chronic kidney disease – ICD-10 codes** | |
| CKD, stage 3 | N18.3 |
| CKD, stage 4 | N18.4 |
| CKD, stage 5 | N18.5 |
| ESRD | N18.6 |
| CKD unspecified | N18.9 |

## Example of the time-updating methodology used during data structuring

Fig. S1 (Additional File 2) demonstrates the time-updating methodology used in the study by summarising the experience of a hypothetical patient. As can be seen, the hypothetical patient had two serum potassium measurements (K1 and K2), three estimated glomerular filtration rate (eGFR) readings (E1, E2 and E3), and one major adverse cardiac event (MACE) during the follow-up period. In the first pass of the analysis, the patient’s first and second intervals began on the dates of serum potassium measurements K1 and K2, respectively. The time between the index date and the start of Interval 1 was ignored, as the patient did not have a serum potassium measurement before K1 (including prior to their index date). The patient’s serum potassium concentration during Interval 1 (time between K1 and K2) was assumed to be consistent with measurement K1, and with measurement K2 during Interval 2 (time between K2 and end of follow-up).

The patient’s eGFR during each interval was assumed to be constant with that observed at the most recent reading at the start of the interval. Therefore, eGFR was assumed to be consistent with measurements E1 and E3 during Interval 1 and Interval 2, respectively. Had reading E3 not been taken, eGFR for Interval 2 would have been assumed to be consistent with measurement E2, and had reading E2 not been taken, eGFR for Interval 2 would have been assumed to be consistent with measurement E1.

The MACE occurred during Interval 2; therefore, Interval 1 did not contribute any events to the incident rate for MACE, while Interval 2 contributed one event (with respective exposure times equal to duration of the intervals in patient-years). In the second pass of the analysis, multiple imputation was conducted on baseline clinical variables. As this patient did not have a serum potassium measurement or an eGFR reading prior to index (K0 and E0, respectively), both variables were imputed with statistical estimates using the R package ‘mice’, and the time between the patient’s index date and their first serum potassium measurement (Interval 0) would then be included in the analysis. The patient’s serum potassium and eGFR in Interval 0 were assumed constant at K0 and E0 respectively, with all subsequent time-updating remaining unchanged from the first pass of the analysis.

**Table S2** Incidence of death, major adverse cardiac event and renin-angiotensin-aldosterone system inhibitor discontinuation observed in chronic kidney disease patients, stratified by serum potassium category

| **Variable** | **All** | **Serum potassium at baseline (mmol/L)^a^** | | | | | | |
| --- | --- | --- | --- | --- | --- | --- | --- | --- |
|  |  | **<3.5** | **3.5 to <4.0** | **4.0 to <4.5** | **4.5 to <5.0** | **5.0 to <5.5** | **5.5 to <6.0** | **≥6.0** |
| Number of patients | 191,964 | 3,635 | 17,662 | 50,065 | 48,543 | 18,454 | 4,250 | 1,026 |
| Number of patient-years | 952,501 | 13,390 | 103,404 | 323,766 | 324,011 | 123,523 | 22,455 | 3,090 |
| **Death** | | | | | | | | |
| Number of events | 44,961 | 1,823 | 5,852 | 13,439 | 13,677 | 6,573 | 2,033 | 694 |
| Rate per 1,000 patient-years (95% CI) | 47  (47, 48) | 136  (130, 143) | 57  (55, 58) | 42  (41, 42) | 42  (42, 43) | 53  (52, 55) | 91  (87, 95) | 225  (208, 242) |
| **MACE** | | | | | | | | |
| Number of events | 80,038 | 1,556 | 9,174 | 25,915 | 26,659 | 10,739 | 2244 | 340 |
| Rate per 1,000 patient-years (95% CI) | 84  (83, 85) | 116  (111, 122) | 89  (87, 91) | 80  (79, 81) | 82  (81, 83) | 87  (85, 89) | 100  (96, 104) | 110  (99, 122) |
| **RAASi discontinuation** | | | | | | | | |
| Number of events | 75,488 | 1,219 | 7,644 | 23,908 | 26,265 | 12,176 | 3,292 | 984 |
| Rate per 1,000 patient-years (95% CI) | 79  (79, 80) | 91  (86, 96) | 74  (72, 76) | 74  (73, 75) | 81  (80, 82) | 99  (97, 100) | 147  (142, 152) | 318  (299, 339) |
| *CI: confidence interval; MACE: major adverse cardiac event; RAASi: renin-angiotensin-aldosterone system inhibitor.*   1. Stratified event incidence include only those patients with an observed serum potassium measurement recorded within +3 months of the index date. | | | | | | | | |

**Table S3** Model output for final risk equations, re-estimated using patient-intervals restricted to a maximum duration of 30 days

| **Explanatory variable** | **Estimate** | **SE** | ***t* statistic** | **P-value** |
| --- | --- | --- | --- | --- |
| **Incidence of death** | | | | |
| Constant | -3.1816 | 0.3478 | -9.15 | <0.0001 |
| Serum potassium: <3.5 mmol/L | 1.2621 | 0.0476 | 26.49 | <0.0001 |
| Serum potassium: 3.5 to <4.0 mmol/L | 0.3750 | 0.0369 | 10.15 | <0.0001 |
| Serum potassium: 4.0 to <4.5 mmol/L | -0.0057 | 0.0300 | -0.19 | 0.3917 |
| Serum potassium: 5.0 to <5.5 mmol/L | 0.2634 | 0.0349 | 7.54 | <0.0001 |
| Serum potassium: 5.5 to <6.0 mmol/L | 0.7059 | 0.0483 | 14.61 | <0.0001 |
| Serum potassium: ≥6.0 mmol/L | 1.4800 | 0.0632 | 23.40 | <0.0001 |
| Age at baseline (years) | 0.0612 | 0.0015 | 40.02 | <0.0001 |
| Gender at baseline: Female | -0.2844 | 0.0236 | -12.05 | <0.0001 |
| Smoker at baseline: Yes | 0.3154 | 0.0330 | 9.55 | <0.0001 |
| Time with CKD (years) | 0.0002 | 0.0000 | 14.27 | <0.0001 |
| Time-updated eGFR (mL/min/1.73m^2^; truncated at 60 mL/min/1.73m^2^) | -0.0283 | 0.0011 | -25.75 | <0.0001 |
| Time-updated prescribed RAASi: Yes | -1.8988 | 0.0308 | -61.60 | <0.0001 |
| Time-updated history of HF: Yes | 0.8068 | 0.0390 | 20.67 | <0.0001 |
| History of diabetes at baseline: Yes | 0.2184 | 0.0339 | 6.43 | <0.0001 |
| History of cancer at baseline: Yes | 0.4776 | 0.0326 | 14.65 | <0.0001 |
| History of PVD at baseline: Yes | 0.1135 | 0.0613 | 1.85 | 0.0717 |
| History of dementia at baseline: Yes | 0.5262 | 0.0573 | 9.19 | <0.0001 |
| History of MACE at baseline: Yes | 0.2246 | 0.0281 | 8.00 | <0.0001 |
| Natural logarithm of baseline BMI (kg/m^2^) | -0.7687 | 0.0881 | -8.73 | <0.0001 |
| Natural logarithm of baseline haemoglobin (g/cL) | -0.7698 | 0.0983 | -7.83 | <0.0001 |
| Prescribed diuretics ± 3 months of baseline: Yes | 0.2493 | 0.0249 | 9.99 | <0.0001 |
| Prescribed bronchodilators ± 3months of baseline: Yes | 0.2365 | 0.0324 | 7.31 | <0.0001 |
| Prescribed insulin ± 3 months of baseline: Yes | 0.1737 | 0.0698 | 2.49 | 0.0180 |
| Prescribed statins ± 3 months of baseline: Yes | -0.1880 | 0.0247 | -7.62 | <0.0001 |
| **Incidence of MACE** | | | | |
| Constant | -4.7431 | 0.1417 | -33.47 | <0.0001 |
| Serum potassium: <3.5 mmol/L | 0.4717 | 0.0552 | 8.54 | <0.0001 |
| Serum potassium: 3.5 to <4.0 mmol/L | 0.2280 | 0.0285 | 7.99 | <0.0001 |
| Serum potassium: 4.0 to <4.5 mmol/L | 0.0818 | 0.0200 | 4.09 | 0.0001 |
| Serum potassium: 5.0 to <5.5 mmol/L | -0.0510 | 0.0253 | -2.02 | 0.0522 |
| Serum potassium: 5.5 to <6.0 mmol/L | -0.0307 | 0.0465 | -0.66 | 0.3211 |
| Serum potassium: ≥6.0 mmol/L | 0.2557 | 0.0925 | 2.77 | 0.0087 |
| Age at baseline (years) | 0.0402 | 0.0011 | 37.23 | <0.0001 |
| Gender at baseline: Female | -0.2683 | 0.0206 | -13.02 | <0.0001 |
| Smoker at baseline: Yes | 0.0040 | 0.0293 | 0.14 | 0.3952 |
| Time with CKD (years) | 0.0002 | 0.0000 | 19.16 | <0.0001 |
| Time-updated eGFR (mL/min/1.73m^2^) | -0.0038 | 0.0009 | -4.25 | 0.0001 |
| History of diabetes at baseline: Yes | -0.1267 | 0.0290 | -4.36 | <0.0001 |
| History of MACE at baseline: Yes | 0.7120 | 0.0234 | 30.44 | <0.0001 |
| History of rheumatologic disease at baseline: Yes | -0.1330 | 0.0502 | -2.65 | 0.0119 |
| History of CPD at baseline: Yes | 0.2361 | 0.0308 | 7.67 | <0.0001 |
| Natural logarithm of baseline total cholesterol (mmol/L) | -0.1032 | 0.0529 | -1.95 | 0.0617 |
| Prescribed CCBs ± 3 months of baseline: Yes | 0.0389 | 0.0208 | 1.87 | 0.0697 |
| Prescribed insulin ± 3 months of baseline: Yes | 0.2125 | 0.0583 | 3.64 | 0.0005 |
| Prescribed beta blockers ± 3 months of baseline: Yes | 0.3044 | 0.0214 | 14.21 | <0.0001 |
| **Incidence of RAASi discontinuation** | | | | |
| Constant | -0.7501 | 0.0410 | -18.29 | <0.0001 |
| Serum potassium: <3.5 mmol/L | 0.2867 | 0.0526 | 5.45 | <0.0001 |
| Serum potassium: 3.5 to <4.0 mmol/L | 0.0349 | 0.0275 | 1.27 | 0.1786 |
| Serum potassium: 4.0 to <4.5 mmol/L | -0.0432 | 0.0195 | -2.22 | 0.0342 |
| Serum potassium: 5.0 to <5.5 mmol/L | 0.1763 | 0.0226 | 7.80 | <0.0001 |
| Serum potassium: 5.5 to <6.0 mmol/L | 0.6437 | 0.0318 | 20.26 | <0.0001 |
| Serum potassium: ≥6.0 mmol/L | 1.4750 | 0.0466 | 31.65 | <0.0001 |
| Gender at baseline: Female | -0.0715 | 0.0157 | -4.57 | <0.0001 |
| Time with CKD (years) | 0.0000 | 0.0000 | -4.21 | 0.0001 |
| Time-updated eGFR (mL/min/1.73m^2^; truncated at 60 mL/min/1.73m^2^) | -0.0275 | 0.0007 | -39.14 | <0.0001 |
| History of diabetes at baseline: Yes | 0.0625 | 0.0208 | 3.00 | 0.0045 |
| History of rheumatologic disease at baseline: Yes | -0.1033 | 0.0369 | -2.80 | 0.0079 |
| History of MACE at baseline: Yes | 0.1106 | 0.0201 | 5.51 | <0.0001 |
| Prescribed diuretics ± 3 months of baseline: Yes | 0.1311 | 0.0160 | 8.19 | <0.0001 |
| Prescribed insulin ± 3 months of baseline: Yes | 0.0366 | 0.0399 | 0.92 | 0.2617 |
| Prescribed CCBs ± 3 months of baseline: Yes | 0.1175 | 0.0164 | 7.17 | <0.0001 |
| *BMI: body mass index; CCB: calcium-channel blocker; CKD: chronic kidney disease; CPD: chronic pulmonary disease; eGFR: estimated glomerular filtration rate; HF: heart failure; MACE: major adverse cardiac event; PVD: peripheral vascular disease; RAASi: renin-angiotensin-aldosterone system inhibitor; SE: standard error.* | | | | |
