# Supplementary figures and images for: Serum potassium as a predictor of adverse clinical outcomes in patients with chronic kidney disease: new risk equations using the UK clinical practice research datalink
Source: BMC Nephrol. 2018 Aug 22;19:211. doi: 10.1186/s12882-018-1007-1 (PMC6106824; doi:10.1186/s12882-018-1007-1)

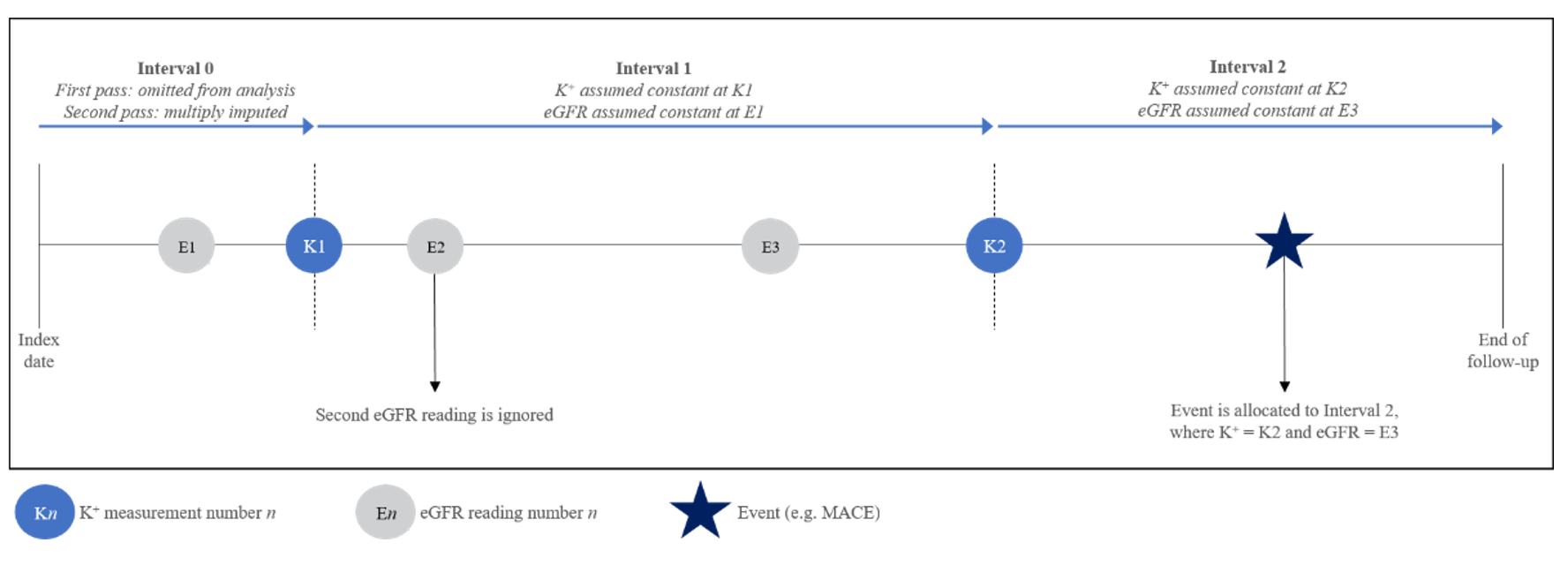

Supplement: Supplementary file 2 — Figure S1. Illustrative example of time-updated patient-intervals based on timing of serum potassium measurements. eGFR: estimated glomerular filtration rate; K+: serum potassium; MACE: major adverse cardiac event. (TIF 260 kb) [file 12882_2018_1007_MOESM2_ESM.tif]

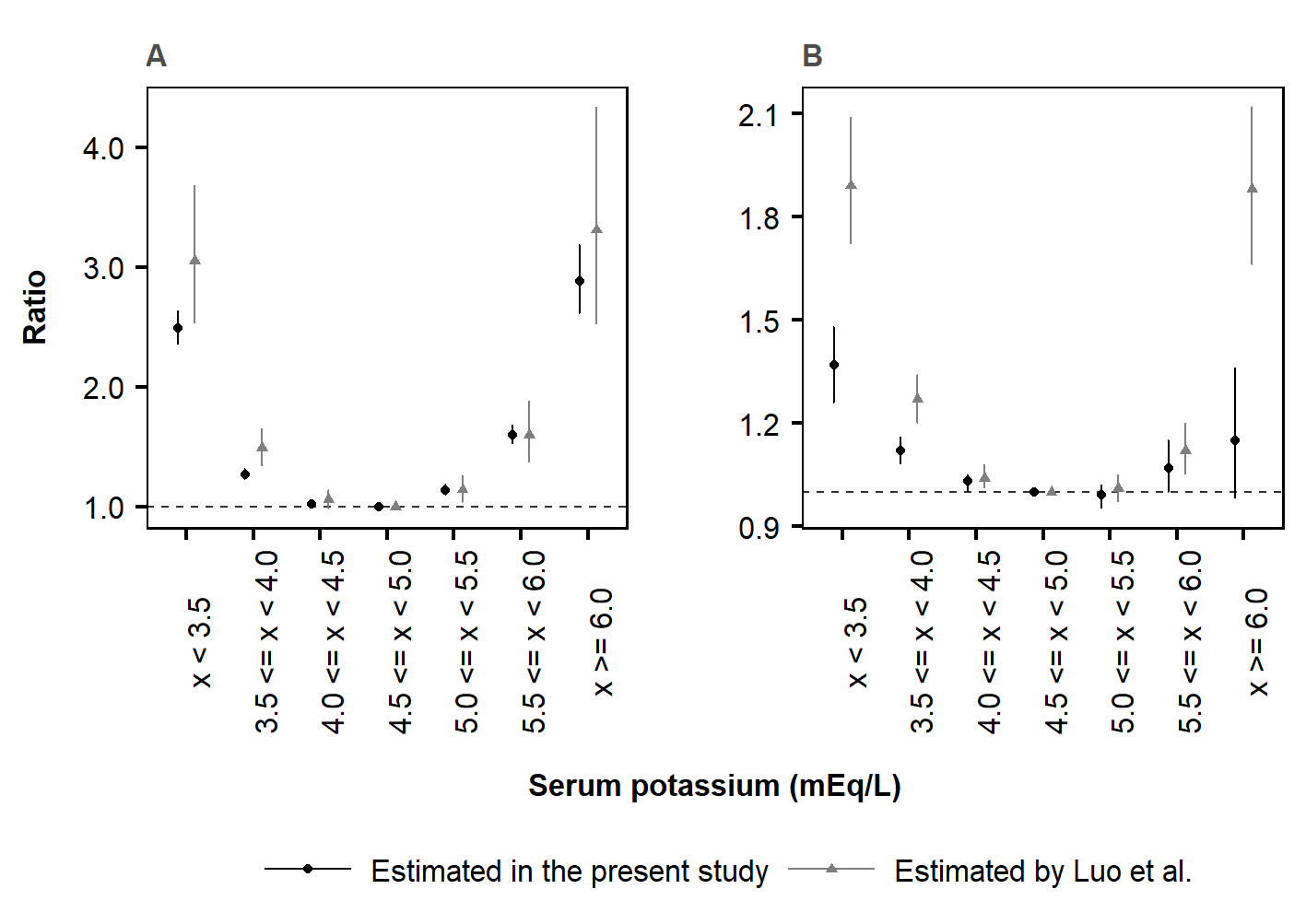

Supplement: Supplementary file 3 — Figure S2. Validation of adjusted incident rate ratios for death (A) and MACE (B) as a function of serum potassium against those published by Luo et al. Black: estimated in the present study (UK CKD stage 3+ patients); grey: estimated by Luo et al. (US CKD stage 3+ patients). Error bars represent 95% confidence intervals. (TIFF 41 kb) [file 12882_2018_1007_MOESM3_ESM.tiff]

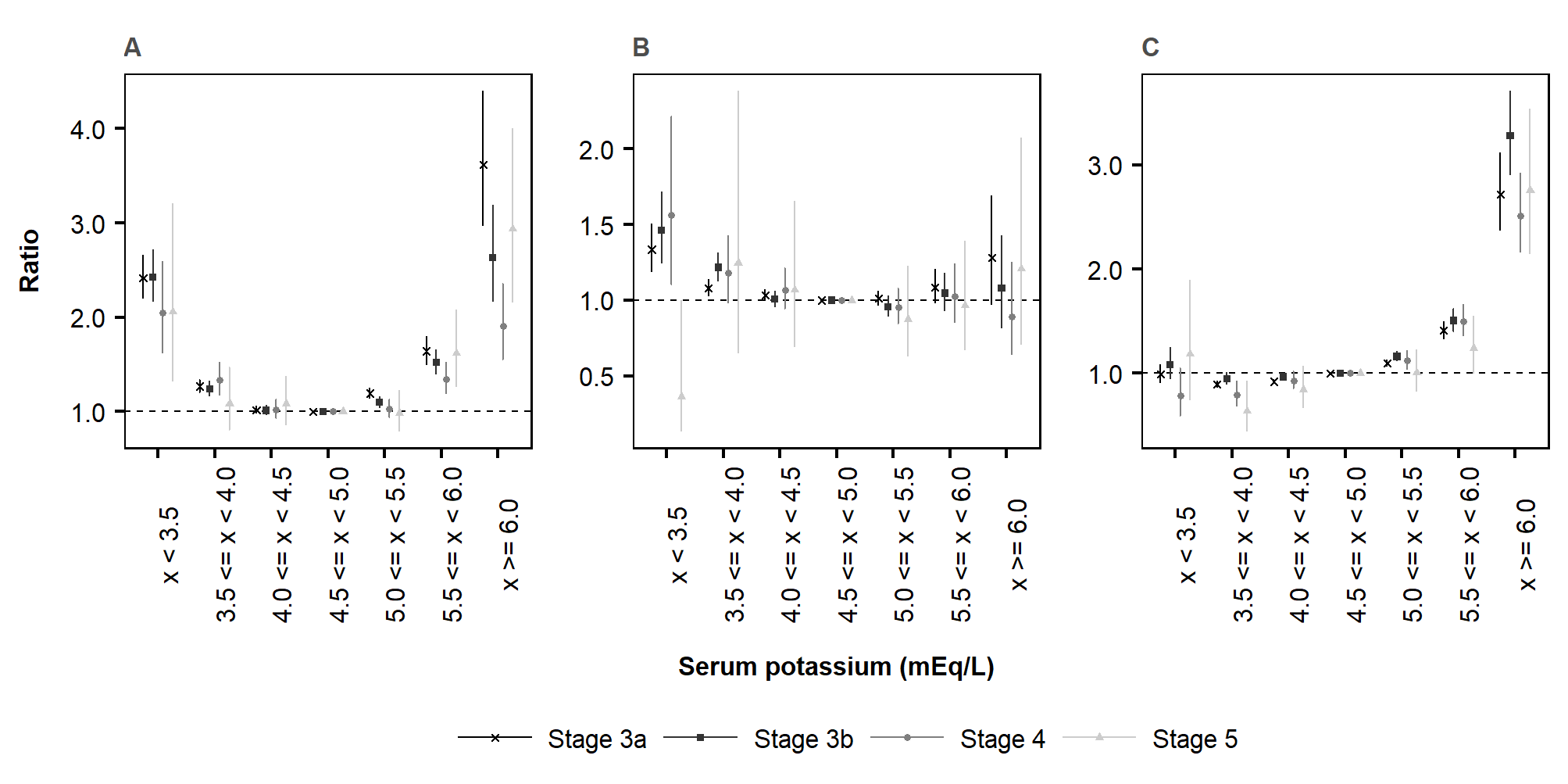

Supplement: Supplementary file 4 — Figure S3. Adjusted incident rate ratios for death (A), MACE (B) and RAASi discontinuation (C) as a function of serum potassium in patients stratified by CKD stage. Black: CKD 3a patients; dark grey: CKD 3b patients; mid-grey: CKD 4 patients; light grey: CKD 5 patients. Incident rate ratios were adjusted to account for confounding patient demographics, clinical histories and comorbidities, clinical measurements, and medication usage, as reported in Table 4. CKD: chronic kidney disease; MACE: major adverse cardiac event; RAASi: renin-angiotensin-aldosterone system inhibitor. Error bars represent 95% confidence intervals. (TIFF 63 kb) [file 12882_2018_1007_MOESM4_ESM.tiff]
